# Practical High-Throughput Method to Screen Compounds for Anthelmintic Activity against *Caenorhabditis elegans*

**DOI:** 10.3390/molecules26144156

**Published:** 2021-07-08

**Authors:** Aya C. Taki, Joseph J. Byrne, Peter R. Boag, Abdul Jabbar, Robin B. Gasser

**Affiliations:** 1 Department of Veterinary Biosciences, Faculty of Veterinary and Agricultural Sciences, Melbourne Veterinary School, The University of Melbourne, Parkville, VIC 3010, Australia; aya.taki@unimelb.edu.au (A.C.T.); byrnej1@unimelb.edu.au (J.J.B.); jabbara@unimelb.edu.au (A.J.); 2 Department of Biochemistry and Molecular Biology, Monash Biomedicine Discovery Institute, Monash University, Clayton, VIC 3800, Australia; peter.boag@monash.edu

**Keywords:** high throughput screening, *Caenorhabditis elegans*, phenotypic screen, motility, infrared light-interference, anthelmintic

## Abstract

In the present study, we established a practical and cost-effective high throughput screening assay, which relies on the measurement of the motility of *Caenorhabditis elegans* by infrared light-interference. Using this assay, we screened 14,400 small molecules from the “HitFinder” library (Maybridge), achieving a hit rate of 0.3%. We identified small molecules that reproducibly inhibited the motility of *C. elegans* (young adults) and assessed dose relationships for a subset of compounds. Future work will critically evaluate the potential of some of these hits as candidates for subsequent optimisation or repurposing as nematocides or nematostats. This high throughput screening assay has the advantage over many previous assays in that it is cost- and time-effective to carry out and achieves a markedly higher throughput (~10,000 compounds per week); therefore, it is suited to the screening of libraries of tens to hundreds of thousands of compounds for subsequent evaluation and development. The present phenotypic whole-worm assay should be readily adaptable to a range of socioeconomically important parasitic nematodes of humans and animals, depending on their dimensions and motility characteristics in vitro, for the discovery of new anthelmintic candidates. This focus is particularly important, given the widespread problems associated with drug resistance in many parasitic worms of livestock animals globally.

## 1. Introduction

The free-living nematode *Caenorhabditis elegans* is a particularly useful model for fundamental studies of metazoan organisms, in general, and for drug discovery and repurposing. This worm has been used quite extensively for investigating the nematocidal/nematostatic effects of toxins and natural or synthetic compounds [1,2,3]. *C. elegans* is easy to culture in the laboratory in large numbers at low cost and can be cryopreserved; it is a small worm (0.6 to 1 mm in length) and, thus, amenable to large-scale screening platforms (e.g., in 384- or 1536-well plate formats; ref. [4]). Other key advantages are the vast molecular, genetic and genomic resources, tools and extensive online information available to study its physiology and biochemistry and gene functions [5] and to identify new drug targets [6,7]. Although it appears that no anthelmintic compound discovered using *C. elegans* as a model has yet reached the clinical phase of development, some compounds that kill *C. elegans* are likely to be effective against other parasitic nematodes when compared with randomly tested molecules [8]. Therefore, the primary screening of compounds on *C. elegans* to precede or complement screening on parasitic nematodes (various developmental stages) to confirm anthelmintic activity, followed by evaluations of toxicity on cells, tissues or vertebrates/mammals can be advantageous [9]. Using such a complementary approach for drug discovery provides a foundation for ensuing studies of the modes of action, metabolisation and detoxification of anthelmintic compounds and for understanding drug resistance mechanisms in a nematode [2,7].

Advances in imaging platforms, computer algorithms and workflows have made this ‘model organism’ a useful option for high throughput screening (e.g., [3,8,10,11]). A range of different optical imaging platforms, including the ‘complex object parametric analyzer and sorter’ (COPAS) Biosort; field-of-view ‘nematode tracking platform’ (WF-NTP); and the ‘invertebrate automated phenotyping platform’ (INVAPP), as well as high-content imaging platforms integrated with microfluidic chips, have been developed [11,12,13,14,15,16]. However, some of these platforms demand significant technical knowledge, skill and experience to set-up and calibrate, in order to achieve repeatable/reproducible results for routine use, such that “off-the-shelf”, commercially available systems can present advantages in terms of cost and time. For example, measuring motility via electrical impedance using the xCELLigence cell monitoring device [17] or with infrared light beam interference using a ‘worm tracking’ system [18,19] has shown promise for developing medium to high throughput techniques; however, a limitation of the latter approach [18,19] is that, usually, interference is measured over an extended period of hours (3 h to 17.5 h) [19,20,21,22,23], which can result in a substantial constraint on throughput. In the present study, we build on previous work of this ‘worm tracking’ system to establish a practical high throughput phenotypic screening platform using *C. elegans* that overcomes this limitation and is amenable to large-scale screens in an academic or industry context.

## 2. Results and Discussion

The present phenotypic screening assay is practical, quantitative and semi-automated. It is now being used routinely in our laboratory, and can achieve a throughput of ~10,000 compounds per week. Central to achieving this throughput has been a clear understanding of technical aspects of WMicroTracker ONE system and the settings (i.e., Modes) available. While setting up the assay, we discovered that the distinct measurement modes (settings) have a profound effect on “activity counts” (measurement of motility), and, from a critical appraisal of the literature, it seems that the WMicroTracker system has often been used without careful consideration of the settings required for screening (e.g., [19,20,21,22,23]). In this study, we learnt that Mode 0 (default) allows the measurement of movement within each well of each plate in a sliding time-window for the subsequent normalisation of data. In our experience, using this setting to measure nematode movement yields very low “activity counts” from individual wells and was, for us, unsuited for high throughput screening using a short data acquisition period. By contrast, Mode 1 constantly records activity counts (all movement) and gives a representative, quantitative measurement of motility, yielding high “activity counts”. By using Mode 1, we achieved a high throughput by being able to capture larval motility within the data acquisition period of 15 min, rather than a period of ≥3 h [19,20,21,22,23].

The motility of worms is recorded simultaneously within individual wells of the 384-well plate by interference of the infrared light beam through each well; this interference is recorded as “activity counts” (translating to motility) [18]. Measuring the motility of 50 worms within individual wells reduces experimental bias that is likely to occur when lower or higher numbers are used, and normalises the motility recorded within each well. The assay relies on the use of fresh L4s, which are readily produced immediately prior to the commencement of a screen. A critical aspect in setting up a screen is ensuring consistency in the number of worms aliquoted per well within and among plates [4]. For *C. elegans* L4s, this consistency was achievable only using both low-retention pipette tips and LB* as the suspension/dispensing medium to overcome the problem of L4s adhering to surfaces (tubes, tips and well-walls). The favourable and consistent Z’-factor (≥0.7) and signal to background (S/B) ratio (>200) show a sound performance of the assay.

**Figure 1:**
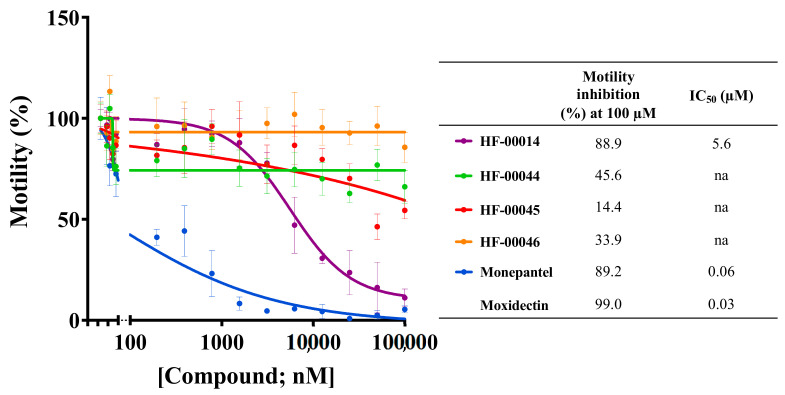
Dose-response curves for hit compound HF-00014, two positive control compounds (monepantel and moxidectin) and three non-hit compounds (HF-00044, HF-00045 and HF-00046; negative reference controls) assessed in 96-well plates for the inhibition of motility of young adult *Caenorhabditis elegans* at 40 h. Each data point represents three replicates, with the mean ± standard error of the mean (SEM) values indicated. As expected, compound HF-00014, identified in the primary screen against *C. elegans* (in the transition from L4 to the adult stage) and achieving 83% motility inhibition (see Table 1), exhibited a dose-dependent effect. IC_50_ values were 0.06 and 0.03 for monepantel and moxidectin, respectively, and 5.6 µM for HF-00014. na = not applicable.

Using this assay, we rapidly screened the 14,400 compounds from the HitFinder library at a concentration of 20 µM for activity on *C. elegans*. At 40 h, we identified 43 compounds that reduced worm motility by ≥70% (Table 1), equating to an overall “hit rate” of 0.3%. Based on compound availability, we then selected a subset of compounds for further evaluation. Dose-response evaluations of HF-00014 showed a reproducible motility reduction (40 h) and an IC_50_ value of 5.6 µM, and no activity for any of the three control compounds (HF-00044, HF-00045 and HF-00046) that had not been identified as hits in the primary screen (Figure 1). As there is no previous report of any of the 43 hit compounds having been tested against a nematode, current work is focused on (i) assessing the toxicity of the most promising compounds, including HF-00014 (Table 1), on HepG2 human hepatoma cells, (ii) establishing dose-response relationships for all of them, and (iii) evaluating their cellular and molecular effects on *C. elegans* tissues [24], as a basis for future work to undertake medicinal chemistry and structure-activity relationship (SAR) studies of the most promising scaffolds/entities.

In conclusion, the screening platform established is practical and straight-forward to set up, costing ~US$65,000, with the majority of this expense relating to the purchase of two WMicroTracker ONE instruments (at US $15,000 each), a semi-automated liquid handling robot (at US $30,000) and an incubator (e.g., Heratherm IMP180, Thermo Fisher Scientific, Waltham, MA, USA; at US $5000) for the culturing of *C. elegans*. This system—now routinely used in our laboratory—meets a high standard and is suited for large-scale screens to identify new chemical entities with nematocidal or nematostatic activities [25]. Screening on *C. elegans* provides the exciting prospect of eventually exploring the mechanisms of action of lead candidates and the development of resistance to them in vitro, particularly given the availability of extensive resources and functional genomics platforms for this free-living nematode. Our plan now is to expand the use of the present screening platform to parasitic worms of socioeconomic importance, including the barber’s pole worm (*Haemonchus contortus*), *Ascaris* and/or hookworms. This focus is of paramount importance, given the widespread problems with anthelmintic resistance in nematode populations of livestock animals.

## 3. Materials and Methods

### 3.1. Preparation of C. elegans Larvae for Screening

A wildtype *Caenorhabditis elegans* strain (Bristol N2) was maintained in the laboratory under standard conditions at 20 °C on nematode growth medium (NGM) agar plates [25], seeded with *Escherichia coli* OP50 as a food source. This *C. elegans* strain was grown and maintained according to well-established protocols [26,27].

*C. elegans* was synchronised using an established method [28]. Gravid adult worms were collected from NGM plates and washed in sterile M9 buffer and then treated with 1% bleach for 4–9 min at room temperature (22–24 °C) to release eggs [26]. Eggs were immediately collected, washed four times in M9 buffer by centrifugation at 500× *g* (2 min) at the same temperature and examined microscopically. Then, eggs were incubated in M9 buffer under slight agitation for ~24 h, allowing first-stage larvae (L1s) to hatch and enter diapause. Approximately 36 h before setting up compound-screening, arrested L1s were pipetted on to NGM agar plates (10 cm) previously seeded with 500 µL of *E. coli* OP50 (~1000 larvae per plate). Plates were incubated at 20 °C to allow worms to synchronously develop to fourth-stage larvae (L4s). On the day of compound-screening, fourth-stage larvae (L4s) were collected from culture plates and washed twice in M9 buffer by centrifugation at 500× *g* (2 min) to remove *E. coli* OP50. L4s were immediately suspended in Luria-Bertani broth [29] containing 100 IU/mL of penicillin, 100 µg/mL of streptomycin and 0.25 µg/mL of amphotericin B (Thermo Fisher Scientific, Waltham, MA, USA) (called LB*) and diluted to a worm density of 2500 L4s per mL.

### 3.2. High Throughput Screening of Compounds for Motility Reduction of C. elegans

The “HitFinder” (HF) library, containing 14,400 (synthetic) small molecular compounds, was curated by Maybridge (Thermo Fisher Scientific, Waltham, MA, USA). These compounds were supplied at a concentration of 10 mM in 100% dimethyl sulfoxide (DMSO). Using a semi-automated liquid handling robot (VIAFLO ASSIST PLUS, Integra Biosciences, Zizers, Switzerland), compounds were individually diluted to 20 µM in LB* containing 0.2% (*v*/*v*) DMSO and subsequently dispensed in 20 µL into the wells of sterile 384-well flat bottom microtitre plates (cat. no. 3680; Corning, Corning, NY, USA); 320 compounds were arrayed on each plate, with 16 wells being negative controls (with LB* + 0.2% DMSO) and four wells containing each monepantel (Zolvix; Elanco, NY, USA), moxidectin (Cydectin; Virbac, Carros, France), monepantel/abamectin (Zolvix Plus; Elanco, NY, USA) and compound MIPS-0018666 (abbreviated here as M-666; see [30]) as positive controls (20 µM). Following the dilution and dispensing of compounds into plates, 50 *C. elegans* L4s in 20 μL of LB* were transferred to individual wells; after this step, the final concentrations were 20 µM of the test- or positive-control compound; and 0.2% of DMSO. During dispensing, L4s were maintained in a homogenous suspension using a constant stream of bubbles produced using an aquarium pump (H2Pro, Melbourne, Australia). Plates containing *C. elegans* L4s were placed in an incubator (Heratherm, model no. IMP180, Thermo Fisher Scientific, Waltham, MA, USA) at 20 °C. After 40 h of incubation with compounds (20 μM), the motility of young adult *C. elegans* (following transition from L4) in individual wells of individual plates was measured for 15 min by infrared light beam-interference [18] using a WMicroTracker ONE instrument (Phylumtech, Sunchales, Argentina; details on the instrument and associated technology are available via http://www.phylumtech.com/home/en/home-en/ (accessed on 8 July 2021) employing the Mode 1_threshold average setting (described in the user manual for this instrument). Raw data (i.e., ‘activity counts’) captured were normalised using measurements for the positive (M-666) and negative (LB* + 0.2% DMSO) controls, in order to remove plate-to-plate variation by calculating the percentage of motility using the program GraphPad Prism v.9.1.0 (GraphPad Software, San Diego, CA, USA). A compound was recorded as a “hit” (i.e., active) if it reduced larval motility by ≥70%. The performance of this assay was continually monitored over time using the Z’-factor [31], which was calculated using data for the negative (DMSO) and the M-666 controls on individual plates (*n* = 43). Assays with a ‘sound’ performance achieve a Z’-factor of 0.5 to 1; the present assay consistently achieved ≥0.7. We also measured the S/B ratio [32] using data from the same control wells; this ratio was consistently >200.

### 3.3. Dose-Response Assay

Dose-response relationships were established to estimate IC_50_ values for selected compounds against *C. elegans*. With reference to two positive control compounds (monepantel and moxidectin), IC_50_ values were established for hit compounds by two-fold serial dilution, starting at a concentration of 100 µM (18-points; in 50 μL of LB*; 100 µM to 0.76 nM), in 96-well plates (cat. no. 3596; Corning, Corning, NY, USA) with larvae dispensed in 50 µL at a density of 100 *C. elegans* L4s per well. After incubation at 20 °C, the motility of young adult worms in individual wells was measured at 40 h of incubation with the compound. Compound concentrations were log_10_-transformed, and fitted using a variable slope four-parameter equation, constraining the highest value to 100%, employing a least squares (ordinary) fit model using the GraphPad Prism software v.9.1.0. Compounds were tested in triplicate. For statistical analysis of larval motility, a one-way analysis of variance (ANOVA) with Tukey’s multiple comparison test or an unpaired *t*-test was used to establish statistically significant differences.

## Figures and Tables

**Table 1:** The 43 ‘hit’ compounds identified in the high throughput screen on *Caenorhabditis elegans* in the transition from L4 to the adult stage. Information regarding the compounds (code and chemical name) and the motility inhibition (≥70%) of young adult worms after 40 h of incubation with the test compound (at 20 µM).

Compound Code	Chemical Name	Motility Inhibition (%)
HF-00001	*N*,*N*′-bis(3,4-dichlorophenyl)urea	100
HF-00002	5-(methylthio)thiophene-2-carboxamide	100
HF-00003	N2-[(dimethylamino)methylidene]-5-(2-thienyl)thiophene-2-carbothioamide	100
HF-00004	13*H*-dibenzo[a,i]carbazole	96
HF-00005	{4-amino-3-[(2-morpholinoethyl)sulfanyl]thieno[2,3-c]isothiazol-5-yl}(phenyl)methanone	92
HF-00006	2-{2-[4-(trifluoromethyl)phenyl]hydrazono}malononitrile	89
HF-00007	5-(1,3-thiazol-2-yl)-2-(2-thienyl)-7-(trifluoromethyl)pyrazolo[1,5-a]pyrimidine	88
HF-00008	5-[(2*E*)-3-(6-hydroxy-4-oxo-2-thioxo-1,2,3,4-tetrahydropyrimidin-5-yl)prop-2-enylidene]-2-thioxodihydropyrimidine-4,6(1*H*,5*H*)-dione hydrate	88
HF-00009	3-(4-chlorophenyl)-6-(phenylmethylene)[1,3]thiazolo[2,3-c][1,2,4]triazol-5(6*H*)-one	86
HF-00010	1-[(2-pyridylamino)methyl]pyrrolidine-2,5-dione	86
HF-00011	methyl 2-[(1-naphthylcarbonyl)amino]-3-phenylpropanoate	85
HF-00012	(2,4-difluorophenyl)[5-(2-thienyl)-2-thienyl]methanone	85
HF-00013	2-(3-benzyl-1-isopropyl-2,4-dioxo-1,2,3,4-tetrahydropyrimidin-5-yl)-1,3-thiazole-4-carbohydrazide	83
HF-00014	5-[4-(2-phenyleth-1-ynyl)phenyl]-1*H*-pyrazole	83
HF-00015	2-{[5-(trifluoromethyl)-2-pyridyl]sulfonyl}ethanohydrazide	81
HF-00016	{5-[5-(2-thienyl)-2-thienyl]-1*H*-pyrazol-1-yl}(4-methoxyphenyl)methanone	81
HF-00017	3,5-di(tert-butyl)-1-methyl-4-nitro-1*H*-pyrazole	81
HF-00018	*N*′-[(5-methyl-3-phenyl-4-isoxazolyl)carbonyl]-3-(2-thienyl)-1,2,4-oxadiazole-5-carbohydrazide	81
HF-00019	9-chloro-12*H*-benzo[5,6][1,4]thiazino[2,3-b]quinoxaline	80
HF-00020	4-phenanthro[9,10-e][1,2,4]triazin-3-ylmorpholine	80
HF-00021	1-[2-nitro-4-(trifluoromethyl)phenyl]piperidine	79
HF-00022	ethyl 5-amino-1-[4-(2,5-dimethyl-1*H*-pyrrol-1-yl)benzoyl]-1*H*-pyrazole-4-carboxylate	79
HF-00023	2-methyl-1-phenylpropan-1-one oxime	78
HF-00024	7*H*-naphtho[1,8-bc]acridin-7-one	77
HF-00025	2,6-di[(2-pyridylthio)methyl]pyridine	77
HF-00026	3-phenyl-2,3-dihydro-1,3-benzothiazol-2-imine	75
HF-00027	4-(tert-butyl)-*N*-(2-cyano-3-fluorophenyl)benzamide	75
HF-00028	di(2,4-difluorophenyl) benzene-1,3-disulfonate	74
HF-00029	cyclopentane-1,2-dicarboxylic acid	74
HF-00030	5,7-dimethyl[1,2,4]triazolo[1,5-a]pyrimidine-2-thiol	74
HF-00031	*N*-(2-{4-[4-(trifluoromethyl)-2-pyrimidinyl]piperazino}ethyl)benzamide	74
HF-00032	[3-(2-chlorophenyl)-5-methylisoxazol-4-yl](4-fluorophenyl)methanone	73
HF-00033	2-({2-[4-(1,3-benzothiazol-2-yl)anilino]-2-oxoethyl}sulfanyl)acetic acid	73
HF-00034	3-(2,2-dimethyl-3,4-dihydro-2*H*-chromen-6-yl)-1-[(4-methoxyphenyl)sulfonyl]-1*H*-pyrazole	73
HF-00035	2-benzo[b]thiophen-3-yl-3-(4-fluorophenyl)acrylonitrile	73
HF-00036	diethyl 4-[(3-methoxyphenyl)thio]pyridine-2,6-dicarboxylate	72
HF-00037	*N*1-(3-fluorophenyl)-2-{[5-(4-nitrophenyl)-2-furyl]methylidene}hydrazine-1-carbothioamide	72
HF-00038	4-bromo-2-(4-bromoanilino)-1,2-dihydroisoquinolin-1-one	72
HF-00039	2-chloro-4-fluoro-*N*-(4-oxo-4H-thieno[3,2-d][1,3]thiazin-2-yl)benzamide	72
HF-00040	*N*1-[3-(trifluoromethyl)phenyl]-2-(3,3-diethoxypropanoyl)hydrazine-1-carboxamide	72
HF-00041	2-(1*H*-indol-3-yl)-3-[4-(trifluoromethyl)phenyl]acrylonitrile	72
HF-00042	ethyl 3-amino-4-(methylamino)benzoate	71
HF-00043	2-(3-methoxybenzyl)-5-[5-(2-thienyl)-2-thienyl]-1,3,4-oxadiazole	70

## Data Availability

Data are within the article.

## Sample Availability

Samples of the compounds are not available from the authors.
